# Total Knee Arthroplasty and Intra-Articular Pressure Sensors: Can They Assist Surgeons with Intra-Operative Decisions?

**DOI:** 10.1007/s12178-021-09724-5

**Published:** 2021-12-28

**Authors:** Liam Z. Yapp, Patrick G. Robinson, Nicholas D. Clement, Chloe E. H. Scott

**Affiliations:** 1 Department of Orthopaedics, Deanery of Clinical Sciences, University of Edinburgh, Chancellors Building, 49 Little France Crescent, Edinburgh, EH16 4SB UK; 2 Department of Trauma & Orthopaedic Surgery, Royal Infirmary of Edinburgh, NHS Lothian, 51 Little France Crescent, Edinburgh, EH16 4SY UK

**Keywords:** Knee, Arthroplasty, Soft tissue, Balance, Sensor, Outcome

## Abstract

**Purpose of Review:**

Soft tissue imbalance, presenting as instability or stiffness, is an important cause of revision total knee arthroplasty (TKA). Traditional methods of determining soft tissue balance of the knee lack precision and are not reliable between operators. Use of intra-operative pressure sensors offers the potential to identify and avoid soft tissue imbalance following TKA. This review aims to summarise the literature supporting the clinical indication for the use of intra-articular pressure sensors during TKA.

**Recent Findings:**

Analytical validation studies suggest that intra-operative pressure sensors demonstrate ‘moderate’ to ‘good’ intra-observer reliability and ‘good’ to ‘excellent’ interobserver reliability throughout the flexion arc. However, there are important errors associated with measurements when devices are used out-with the stated guidelines and clinicians should be aware of the limitations of these devices in isolation. Current evidence regarding patient benefit is conflicting. Despite positive early results, several prospective studies have subsequently failed to demonstrate significant differences in overall survival, satisfaction, and patient-reported outcome measures within 1 year of surgery.

**Summary:**

Surgeon-defined soft tissue stability appears to be significantly different from the absolute pressures measured by the intra-operative sensor. Whilst it could be argued that this confirms the need for intra-articular sensor guidance in TKA; the optimal ‘target’ balance remains unclear and the relationship with outcome in patients is not determined. Future research should (1) identify a suitable reference standard for comparison; (2) improve the accuracy of the sensor outputs; and (3) demonstrate that sensor-assisted TKA leads to patient benefit in patient-reported outcome measures and/or enhanced implant survival.

## Introduction

A sensor is broadly defined by the Oxford Dictionary as a ‘*device which detects or measures a physical property and records, indicates or otherwise responds to it*’. Biometric Monitoring Technologies (BioMeTs) are digital medicine devices which process medical or health data captured by mobile sensors [1•]. Using data-driven algorithms, BioMeTs generate measures of behavioural and/or physiological function.

The purpose of any technology used to assist a surgical procedure is to enhance operator precision, reduce the presence of outliers and to improve patient outcome [2]. Rather than relying entirely on subjective interpretation, sensor-assisted surgery aims to provide quantifiable information which can supplement the experience of the surgeon [3].

Although there are examples of biomechanical studies utilising implantable sensors in surgery of the spine, hip, and shoulder [4, 5], few clinical studies involving these devices have been undertaken. Conversely, in recent years there has been an increasing interest in sensor-assisted soft tissue balancing in primary total knee arthroplasty (TKA). Accordingly, this review will focus on the current uses and evidence base surrounding sensor-assisted TKA surgery.

## Background

Arthritis is a major global cause of adult disability and it is reported that over 300 million people are affected by either hip or knee osteoarthritis [6]. The lifetime risk of symptomatic knee osteoarthritis is estimated to be as high as 44.7% [7]. A significant proportion of these patients will require total knee arthroplasty (TKA) if their symptoms of pain and disability are not controlled adequately by simple measures such as activity modification and analgesia [8, 9].

TKA is associated with a successful outcome in the majority of cases; however, approximately one in five patients will not be satisfied following surgery [10, 11]. Many factors influence the outcome of primary knee arthroplasty and may be broadly classified as patient [12–14], surgical [15], or system factors [16, 17]. It is reported that up to 7–24% of revision TKAs are performed for soft tissue imbalance in the form of joint instability [15, 18, 19]. Accordingly, there is increasing focus on techniques that can improve instability-related factors such as overall limb alignment, implant positioning, flexion-extension balance of the knee, and management of the soft tissue envelope [20].

## Rationale for Sensor-Assisted Total Knee Arthroplasty

Insall advocated altering the native knee’s anatomical alignment to pursuing neutral mechanical alignment through positioning the femoral and tibial implants perpendicular to the mechanical axis of the limb [21]. This would distribute the load transmitted through the knee more evenly and avoid complications such as uneven polyethylene wear which can lead to early failure. To achieve this, the tibia is cut in zero degrees to enable the mechanical axis to pass through the centre of the knee joint. In order to balance the flexion gap, the femur is positioned in 3 degree of external rotation to compensate for the relative valgus position of the tibial component.

More recently there has been increasing interest in pursuing a more anatomical, or kinematic, alignment of the knee [22]. This method attempts to recreate the normal tibio-femoral joint articulation and places the tibial and femoral component axes in alignment with the three kinematic axes of the ‘normal’ knee. In theory, kinematic alignment is more patient-specific and should require minimal soft tissue releases as bone cuts are symmetrical for medial and lateral compartments [23]. Using sensor-reported pressures as a primary outcome measure, MacDessi et al. [24] demonstrated in a randomised control trial (RCT) of kinematic versus mechanical alignment that restoring the constitutional alignment with kinematic alignment in TKA resulted in a statistically significant improvement in knee balance with less difference in pressure between medial and lateral compartments and less need for recuts or releases. However, as this technique ignores the overall limb alignment in the coronal plane, there are fears that KA will create varus-valgus outliers and therefore contribute to early failure [25]. Although much research has been undertaken to identify which technique provides the optimal outcome for patients, no convincing conclusions regarding superiority have been drawn [26–28].

Navigation, robotic systems, and patient-specific instruments have added objectivity and precision to help define a more functional alignment and anatomic rotation when performing TKA [23, 29]. However, the concept of soft tissue balance continues to be principally determined by the subjective ‘feel’ of the surgeon [30]. This can be affected by overall surgeon experience, operative technique, and patient-specific variables such as body mass index (BMI), gender, co-morbidity, and relative ligamentous laxity [20]. Whilst many surgeons agree that it is important, the definition of a balanced TKA appears to be contentious and often hard to define [31]. It is perhaps understandable that intra-operative surgeon assessment has been shown to be highly inaccurate and a poor predictor of TKA balance [32•].

Intra-articular sensor-based balancing can provide dynamic, intra-operative feedback for the surgeon regarding overall tibio-femoral contact point, kinematic tracking, and pressure monitoring in areas of peak contact in the medial and lateral compartments of the knee [25, 30, 33]. Compared to traditional gap balancing techniques, sensor-based TKA allows the patella to be reduced during measurements whereas tensiometers do not [34]. This is relevant as it avoids the extensor mechanism acting as a lateral tether, which may inadvertently affect compartmental loads, and enables accurate tibio-femoral tracking. In theory, this data can be used by the operating surgeon to correct soft tissue imbalance in a controlled, targeted, and quantifiable fashion.

## Evaluation of Intra-Operative Sensors Used To Balance TKA

A key element of evaluating a diagnostic test (‘index test’) is to determine its diagnostic accuracy, i.e. the ability of the test to discriminate between patients with and without the target condition [35]. In ideal conditions, assessment of diagnostic accuracy relies upon a ‘gold standard’ reference which can accurately identify whether the target condition is present or not [36]. However, as is the case with many medical conditions, there is no error-free ‘gold standard’ available to determine intra-articular soft tissue knee balance. In such situations, researchers will use the best available method to determine the presence or absence of the target condition, often termed the ‘reference standard’ [37, 38].

The definition of soft tissue balance of the knee is poorly defined with little consensus available in the literature [31]. When the gold standard is absent, and the diagnostic accuracy of the reference standard is unknown, current guidance suggests undertaking alternative methods of assessment such as test validation and analytical sensitivity [37, 38]. Such methods assess how accurate the ‘index test’ is at measuring what it is designed to measure.

All diagnostic tests, including BioMeTs, should be systematically evaluated to verify and validate their findings [37–39]. Goldsack et al. [1•] recently described a three-component framework—verification, analytical validation, and clinical validation (V3)—which can be used to evaluate BioMeTs in digital medicine (Table 1).

**Table 1:** The stages of V3 for a BioMeT (adapted from Goldsack et al. [1•])

Step	Component	Description	Responsibility	Example question
1	Verification	Systematic evaluation of sensor performance and the generated sample-level data against pre-specified criteria.	Manufacturer	Is the raw data from the pressure sensor accurate, precise, and consistent?
2	Analytical validation	Evaluates algorithm performance and ability of BioMeT to measure, detect, or predict physiological metrics	Manufacturer Sponsor Clinical researchers	Does the pressure sensor and processing algorithms provide clinical-grade accuracy of intra-compartmental pressures?
3	Clinical validation	Evaluates whether BioMeT acceptably identifies, measures, or predicts a meaningful clinical, biological, physical, functional state or experience in the stated context of use (which includes a specified population)	Sponsor Clinical researchers	Do intra-compartmental pressures detect soft tissue imbalance of the knee? Does adjustment of intra-compartmental pressures predicts knee joint stability?

## Intra-Operative Technique and Supporting Evidence

To the authors’ knowledge at present, there are two commercially available sensor-guided intra-operative soft tissue balancing technologies for TKA: the VERASENSE Knee System (OrthoSensor, Dania FL, USA) and the eLIBRA Dynamic Knee Balancing System (Synvasive Technology, Zimmer-Biomet, Reno, NV, USA). Both systems employ single-use, modular components which are designed to provide real-time quantifiable feedback during the surgeon’s standard approach to TKA with minimal workflow disruption. The following sections will focus on the analytical and clinical validation of pressure sensors in TKA.

### VERASENSE Knee System (KS)

The VERASENSE KS has been developed with microelectronics embedded into a single-use modular tibial trial component [33]. The VERASENSE KS sensor communicates wirelessly with an OrthoSensor LinkStation which is uploaded with software which can interpret the measurements as compressive loads displayed in pounds (lb) and feeds this back to the operating surgeon using a visual display.

The VERASENSE KS was originally available to be used with three companies’ implants (Stryker: Triathlon®; Zimmer-Biomet: Persona®, NexGen®, and Vanguard®; and Smith & Nephew: Legion® and Journey II®). Following full bony resection of the femur and tibia, the trial components are inserted, and manually assessed by the operating surgeon. A VERASENSE KS component can then replace the trial polyethylene insert and shims are inserted to mimic the desired polyethylene thickness. Upon activation of the component, the tibial component is positioned and held with static pins before the capsule is temporarily closed with clips. The knee is then put through a full range of motion to assess tibial rotation and soft tissue tension. This provides medial and lateral tibio-femoral contact forces and points of contact to be recorded as the knee is passively flexed to 10°, 45°, and 90°. Based on the feedback provided, the operating surgeon can then choose whether soft tissue releases of either the medial or lateral structures or further bony resection are required.

### eLIBRA Dynamic Knee Balancing System (DKBS)

The eLIBRA DKBS is only designed for Zimmer-Biomet knee implants (Vanguard® and Persona®). It has femoral and tibial components which can be adjusted to suit the needs of each patient. The tibial sensor sits beneath the trial tibial component and is attached to a handle with an electronic display. Compressive forces are displayed as units (ranging between 1 and 20) equating to approximately 15N (3.4 lb) per unit.

Unlike the VERASENSE KS, the eLIBRA DKBS is used after the extension gap is produced following distal femoral and proximal tibia resection. The LIBRA Femoral Component is then positioned and secured against the distal femur with respect to the centre of the intercondylar notch and the posterior femoral condyles. Once the LIBRA Femoral Component is satisfactorily positioned, the corresponding sized eLIBRA tibial insert is chosen and the sensor is activated. The patella is then reduced to ensure all dynamic forces are considered and the knee is flexed to 90 degrees. The medial load is then demonstrated by a number on the sensor handle electronic display. The tibial sensor values should range from 4 to 9 (i.e. 13.6–30.6 lb) on the medial side. If the medial loads are lower than 4 (i.e. < 13.6 lb), a thicker tibial insert is required. If the medial load is higher than a 9 (i.e. > 30.6 lb), a thinner insert is required with or without additional soft tissue releases and further tibial resection.

Once the target medial load is achieved, the insert height has been established and attention can be turned to adjustment of the articulating LIBRA femoral component. Using the adjustment mechanism, the lateral posterior femoral condyle is elevated (and thereby externally rotated up to a maximum of 10 degrees) with the intention to equalise the relative forces in the medial and lateral tibio-femoral compartments. Once satisfactorily balanced, the remaining femoral resection is performed, and the procedure completed as per the standard implant surgical technique.

### Analytical Validation—Are the Pressure Sensors Used in TKA Accurate and Reliable**?**

Several cadaveric, biomechanical, and clinical studies have been undertaken investigating the potential utility of pressure sensors in TKA. Collateral ligament and intra-compartment contact forces measured during TKA have shown significant correlation and a linear relationship has been demonstrated throughout the flexion arc [40]. This would suggest that quantitative measurement of condylar contact forces is a suitable alternative for the measurement of soft tissue balance of the knee.

The VERASENSE KS manufacturers advise a measurement range of 5–40 lb force per compartment and a maximum force of 70 lb per compartment. In the event that pressures exceed the maximum value, the device should be removed and re-zeroed prior to reuse. In light of these recommendations, Nicolet-Peterson et al. [41] evaluated the accuracy of tibial contact force measurements and location errors. When computing the worst-case scenario for values out-with the acceptable range of loading, the bias, precision, and root-mean-square error (RMSE) for tibial contact force imbalance were 0 lb, 4.4 lb, and 4.4 lb force respectively. Furthermore, when loading occurred outside the sensing area in one compartment, the error was increased leading to inaccurate tibial contact force and contact location measurements. If an intra-compartmental force difference of less than 15 lb is targeted, then the VERASENSE KS force measurements could exceed this value 16% of the time, despite the knee achieving the target imbalance [41]. This could result in unnecessary soft tissue releases in approximately one in seven patients. The authors suggest that surgeons should be cautious in the interpretation of the VERASENSE KS readings and use it to supplement their clinical experience.

Pressure sensors used in TKA appear to be reliable on repeat testing and between observers. Test-retest load measurements have been shown to be less than 3 lb (1.4 kg) across multiple knees [42]. The intra-observer agreement varied between moderate to good in the majority of measurements, in both a blinded and unblinded setting [43]. However, the lowest level of agreement was observed at 10 degrees of flexion (medial intra-class correlation (ICC) 0.52, 95% CI 0.20–0.74; lateral ICC 0.64, 95% CI 0.38–0.81). Conversely, Thompson et al. [44] demonstrated using Bland-Altman plots that excellent interobserver agreement of VERASENSE measurements was noted at 10 degrees flexion, but noted that this agreement decreased with increasing flexion. However, interobserver agreement showed ICC values ranging between good and excellent reliability for medial compartment pressures measured at 10 degrees (0.93, 95% CI 0.89–0.95), 45 degrees (0.91, 95% CI 0.87–0.93), and 90 degrees of flexion (0.88, 95% CI 0.83–0.91) [44]. Similarly, the ICC for lateral compartment pressure measures was excellent (ICC 0.91, 95% CI 0.87–0.93) at 10 degrees, and good for both 45 degrees (ICC 0.76, 95% CI 0.68–0.82) and 90 degrees flexion (ICC 0.76, 95% CI 0.67–0.82).

Sensor assistance has also improved the understanding of how surgical technique can influence intra-compartmental loads. Manning et al. [45] investigated the role of femoral component position on medial and lateral compartment pressures. These authors found no difference in the loading patterns of either compartment in a neutral knee across the entire flexion arc. Interestingly, whilst an internally rotated femoral component led to very high medial compartment pressures over 60 degrees of flexion without instability, externally rotating the femoral component did not produce a similar outcome in the lateral compartment.

In a cadaveric study utilising computer navigation and sensor assistance, rotation of the tibial component has also been shown to alter the forces through the medial compartment of the knee [46]. These findings suggest that external rotation of the tibial tray could potentially lead to early failure of the medial compartment polyethylene. However, external rotation of the tibial component did not alter knee laxity during flexion when compared with neutral rotation.

Sensor-assisted TKA may also enable clinicians to perform soft tissue releases in a controlled and safe manner. Step-wise puncturing of the medial collateral ligament (MCL) has been shown to significantly reduce the medial compartment pressures and correlated significantly with joint gap measurement [47]. Whether step-wise puncturing alters laxity postoperatively in the longer term remains unclear as this technique is used for arthroscopic meniscal surgery without being complicated by persistent MCL laxity [48].

### Clinical Validation—What Is a Balanced TKA**?**

When adhering to manufacturer guidance, measurement of inter-compartment forces has been shown to be accurate and reliable between users. However, one major issue is how to interpret absolute pressure values and the inter-compartmental difference. The developers of VERASENSE suggest that the knee is considered to be balanced when it satisfies the following criteria [3]: (1) The joint must be stable in the sagittal plane, demonstrated by a stable end-point during application of a posterior drawer test; and (2) the respective compressive loads in the medial and lateral compartments of the knee are below 55 and 45 lb, with an intra-compartmental difference of less than 15 lb.

Gustke et al. [3] reported that this latter cutoff value was chosen based upon previous biomechanical research into intercondylar compartmental pressures [49], and intra-operative observation of ‘experienced’ surgeons following varus-valgus stress testing of the knee of 2 mm using computer-assisted navigation. A further association with postoperative improvement in the original American Knee Society Score (KSS) was reported; however, it was subsequently confirmed that this was a post hoc finding [50].

Meneghini and colleagues [51] attempted to validate this target ligament balance by performing a multi-centre retrospective case-series in which the VERASENSE KS was used to measure intra-compartmental pressures during TKA, but was not used to guide soft tissue balancing. These authors found no association with intra-compartmental force and patient-reported function or satisfaction scores concluding that the ‘less than 15 lb’ cutoff was arbitrary and may not evidence-based.

Meere et al. [52] suggested a balanced approach, using a ratio of the medial force to the total force, aiming for a ratio of 0.5 if both compartments were equally matched. Prior to balancing, the contact force ratio went from 0.49 ± 0.27 to 0.52 ± 0.14. These authors considered a contact force ratio between 0.35 and 0.65 to be ‘acceptable’ based upon subjective assessment of knee balance. Subsequent studies utilising this ratio found no correlation between the contact force ratio and knee society scores of symptoms, satisfaction, and function [53].

Jacobs et al. [54] used the eLIBRA to determine whether symmetrical forces across the medial and lateral compartments had greater correlation with satisfaction and other patient-reported outcome measures (PROMs). These authors converted their results to pounds, thereby enabling their results to be viewed in the context of the VERASENSE KS. They reported a greater proportion of satisfied patients among those who exhibited greater forces in the medial compartment, which may be explained as this is thought to be similar to the pattern of contact forces observed in the native knee [55].

Shelton et al. [56•] looked at the range of published force targets [50–52, 54] and found no association with outcome in patients undergoing kinematically aligned TKA. Despite these findings, the majority of research [24, 42, 43, 57, 58, 59••, 60–62] related to the VERASENSE KS continues to use the cutoff of inter-compartment difference of less than 15 lb [3].

### Clinical Validation—Sensor-Assisted TKA and Outcomes

Surgeon-defined soft tissue stability, either determined by ‘feel’ or through use of a tensiometer during TKA, appears to be significantly different from the absolute pressures measured by the intra-operative sensor [30, 63]. Whilst it could be argued that this confirms the need for intra-operative sensor guidance in TKA, the optimal ‘target’ balance however remains unclear and the relationship with outcome in patients is yet to be determined.

It is hoped that improving soft tissue balance will result in enhanced PROMs and longer duration of implant survival. No difference has been reported in overall complications or revision within 1 year [51, 59••, 64]. To date, there are no studies comparing implant survival of sensor-assisted versus manual TKA beyond 2 years.

Current evidence regarding patient-reported outcomes is conflicting. Two industry-sponsored reports suggested that ‘balanced’ knees were associated with greater improvement in PROMs and satisfaction [50, 65]. However, several retrospective and prospective studies have subsequently failed to demonstrate any significant difference in overall satisfaction, knee-specific outcomes, and general health-related quality of life measures at 6 and 12 months [42, 51, 56•, 59••, 62, 64, 66]. These findings could suggest that the current ‘target’ balance utilised by most authors is either incorrect or at the very least too narrow to identify a difference. In the absence of evidence demonstrating improved implant survival or enhanced PROMs, it is not possible to report on the cost-effectiveness of sensor-assisted TKA.

## Areas for Future Development

There are fundamental elements regarding the diagnostic and clinical validity of commercially available pressure sensors used in TKA which are yet to be determined. Future research should be focussed on the following areas: (1) identification of a suitable reference standard for comparison; (2) improve the accuracy of the sensor outputs; (3) demonstration that sensor-assisted TKA leads to patient benefit in PROMs and/or enhanced implant survival.

## Conclusions

Soft tissue imbalance, presenting as instability or stiffness, is an important cause of revision TKA. Traditional methods of determining soft tissue balance of the knee are not defined and are not reliable between operators. Use of intra-operative sensors offers the potential to identify, predict, and avoid soft tissue imbalance following TKA. To enhance our understanding of the utility of currently available devices, future research should (1) identify a suitable reference standard for comparison; (2) improve the accuracy of the sensor outputs; and (3) demonstrate that sensor-assisted TKA leads to patient benefit in patient-reported outcome measures and/or enhanced implant survival.
